# Transdermal Drug Delivery Systems in Atopic Dermatitis: A Review of Vehicle Innovation and Skin Barrier Challenges

**DOI:** 10.7759/cureus.94120

**Published:** 2025-10-08

**Authors:** Andres D Parga, Natasha Doshi, Riya M Bhat, Toan Vu, Kiratpreet Sraa, Stephanie Casagrande, Rahul Borra

**Affiliations:** 1 Medicine, Hospital Corporation of America (HCA) Florida Healthcare/University of South Florida (USF) Morsani College of Medicine, Brooksville, USA; 2 Medicine, Lake Erie College of Osteopathic Medicine, Bradenton, USA; 3 Internal Medicine, University of Missouri-Kansas City School of Medicine, Kansas City, USA; 4 Medicine, University of Wisconsin School of Medicine and Public Health, Madison, USA; 5 Dermatology, Arizona College of Osteopathic Medicine, Glendale, USA; 6 Medicine, Rutgers Robert Wood Johnson Medical School, Piscataway, USA; 7 Internal Medicine, University of South Florida (USF) Hospital Corporation of America (HCA) Oak Hill Hospital, Brooksville, USA

**Keywords:** atopic dermatitis, emulsions, hydrogels, nanocarriers, nanoparticles, skin barrier dysfunction, solid lipid nanoparticles, topical therapeutics, transdermal drug delivery

## Abstract

Atopic dermatitis (AD) is a chronic, relapsing inflammatory skin condition marked by immune dysregulation, pruritus, and impaired skin barrier function. Effective transdermal drug delivery is essential for therapeutic success but remains hindered by stratum corneum (SC) dysfunction and variable skin permeability. This review examines recent advancements in vehicle formulations designed to enhance transdermal drug delivery, specifically in the context of AD. Peer-reviewed studies published within the last two decades were reviewed using PubMed and Google Scholar (Google LLC, Mountain View, California, United States), focusing on innovations in drug delivery systems, vehicle composition, and clinical applications. Novel delivery platforms such as liposomes, ethosomes, nanoemulsions, polymeric nanoparticles, solid lipid nanoparticles (SLNs), and nanostructured lipid carriers (NLCs) have demonstrated improved percutaneous absorption, increased drug stability, and reduced skin irritation. Additional breakthroughs include biomimetic formulations, occlusive enhancers, and pH-sensitive carriers tailored to the disrupted skin barrier in AD. Despite these advancements, significant challenges remain, including variability in skin hydration, interindividual differences in barrier function, and concerns about long-term safety. Continued research and clinical trials are necessary to validate the efficacy and tolerability of these technologies. Future advances may lie in personalizing transdermal therapies through skin barrier phenotyping, enabling more targeted, tolerable, and effective treatment strategies.

## Introduction and background

Atopic dermatitis (AD) affects an estimated ~2.6% (95% UI 1.9-3.5) of the global population in a given year, though in many higher-income settings 12-month prevalence estimates cluster in the 5-10% range, with especially greater prevalence in children (often > 5-10%) [1]. Beyond its hallmark symptoms of pruritus, erythema, and lichenified lesions, AD is increasingly recognized as a multifactorial disease rooted in both immune system dysregulation and fundamental defects in the epidermal barrier [2]. These disruptions render the skin not only more susceptible to environmental allergens and microbial invasion but also profoundly alter its capacity to absorb and retain therapeutic agents. Topical therapy remains the primary intervention for most patients with AD, despite advances in the immunomodulatory treatments. The rationale is clear: direct, localized delivery of medication offers the potential for rapid symptom control with reduced systemic side effects [3]. However, the very barrier abnormalities that drive disease progression, namely, reduced ceramides, altered lipid architecture, and filaggrin deficiency, also hinder the effectiveness of topical drugs [4,5]. In many cases, poor percutaneous absorption and heightened sensitivity can compromise both the efficacy and tolerability of standard formulations, leading to treatment dissatisfaction and reduced adherence [6]. Recent years have witnessed a surge in research aimed at optimizing transdermal drug delivery in AD. Innovative vehicle technologies, including nanoscale carriers, hydrogels, and film-forming systems, are being engineered to overcome the unique physicochemical and immunological challenges presented by diseased skin [7-11]. These platforms are designed not only to enhance drug penetration through compromised barriers but also to stabilize active compounds and minimize local irritation. This review provides a comprehensive overview of the current landscape in transdermal delivery systems for AD.

Objectives

By reviewing and discussing recent advances in vehicle formulation and their mechanistic and clinical implications, we aim to identify emerging strategies that may bridge the gap between drug development and real-world therapeutic outcomes in AD.

## Review

Methods

This narrative review was conducted to report recent advancements in transdermal drug delivery systems for AD, with a particular focus on vehicle innovations designed to overcome skin barrier dysfunction. A comprehensive literature search was performed using PubMed and Google Scholar (Google LLC, Mountain View, California, United States) databases to identify peer-reviewed articles published between January 2009 and March 2025. Search terms included combinations such as "atopic dermatitis," "transdermal drug delivery," "skin barrier," "nanocarrier," "hydrogel," "film-forming systems," "microneedle," "emollient," and "topical therapy." Additional keywords related to specific delivery vehicles (e.g., "liposomes," "nanoemulsions," "solid lipid nanoparticles," "polymeric nanoparticles," "microemulsions") were also incorporated to maximize sensitivity.

Inclusion criteria comprised original research articles, systematic reviews, meta-analyses, and selected narrative reviews that addressed innovations in vehicle formulation, drug penetration, and clinical outcomes in AD. Studies were included if they evaluated either preclinical (in vitro or animal) models or human clinical populations with AD. Only articles published in English were considered. Studies focusing on drug delivery for other inflammatory skin diseases were excluded unless mechanistic data were directly translatable to AD pathophysiology.

Data extraction emphasized the physicochemical and biological rationale for each delivery system; vehicle composition and mechanism of action; preclinical and clinical evidence of efficacy, safety, and tolerability; and limitations, adverse effects, and barriers to clinical implementation. Particular attention was paid to innovations in barrier compatibility, patient adherence, pediatric application, and the development of personalized or stimuli-responsive vehicles. Where available, reference lists of key articles and recent systematic reviews were hand-searched for additional sources.

All included studies were evaluated for methodological quality, relevance to AD, and clarity in reporting vehicle outcomes. The findings were reported qualitatively, and results were organized thematically according to delivery vehicle category (emollient/occlusive, liposomal/nanocarrier, hydrogel/microemulsion, polymeric/film-forming, and microneedle systems). No statistical meta-analysis was performed due to heterogeneity in study designs, outcome measures, and populations. Instead, the narrative synthesis approach allowed for a comprehensive appraisal of mechanistic advances, clinical evidence, and persistent challenges in the field.

The review also integrated emerging perspectives on future directions, including artificial intelligence-guided personalization and regulatory considerations. All content was reviewed and revised for accuracy by the authors. This methodology was designed to provide an up-to-date, critical overview of transdermal delivery strategies in AD and to guide both clinical practice and future research.

Pathophysiology of skin barrier dysfunction in atopic dermatitis

**Figure 1:**
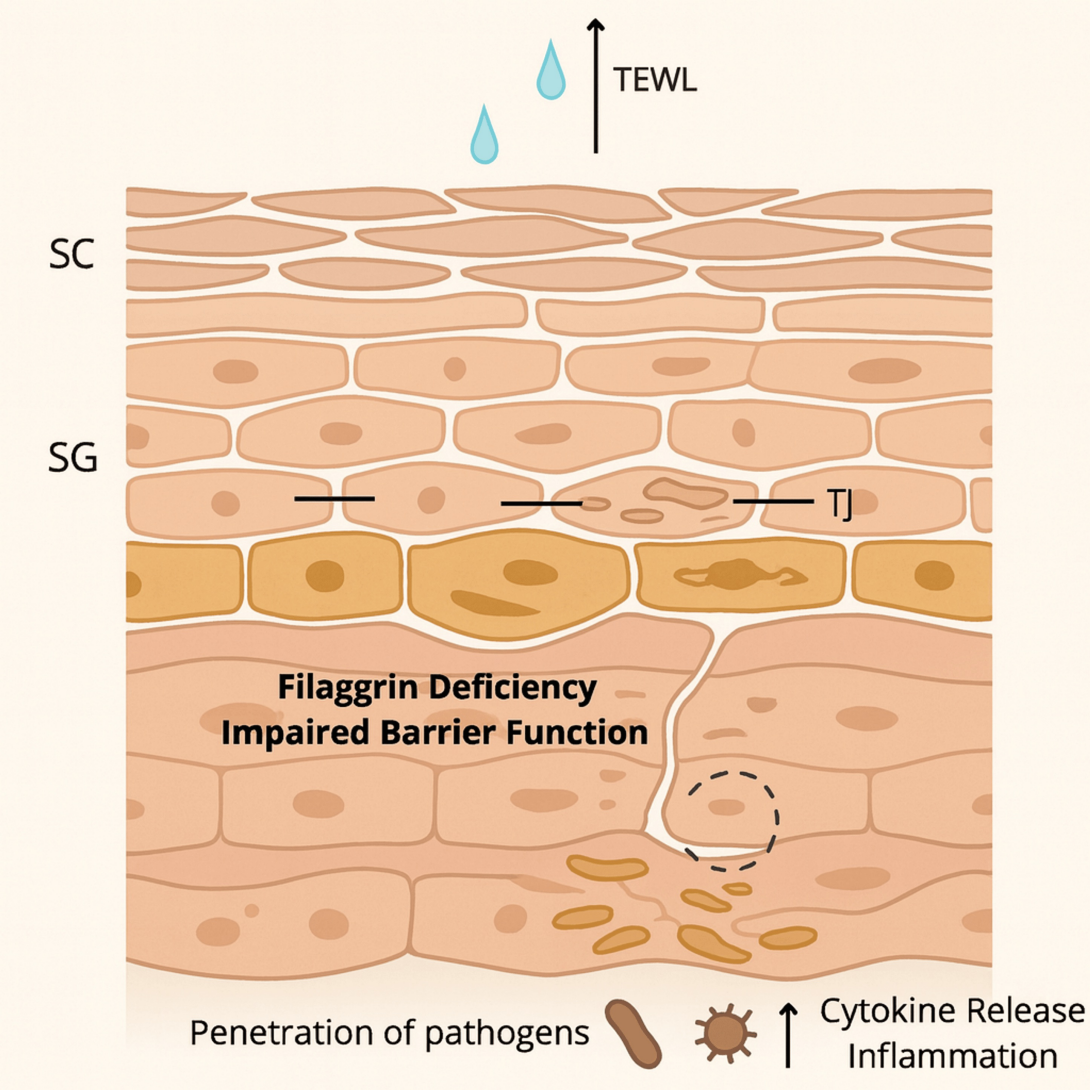
Schematic representation of skin barrier disruption in atopic dermatitis This schematic illustrates key features of epidermal barrier dysfunction characteristic of atopic dermatitis (AD). Filaggrin deficiency and impaired barrier function in the stratum corneum (SC) and stratum granulosum (SG) result in increased transepidermal water loss (TEWL), disrupted tight junctions (TJ), and heightened penetration of pathogens. The compromised barrier facilitates cytokine release and inflammation, contributing to the chronic disease state. Th2 cytokines (↑ IL-4, IL-13) suppress filaggrin and alter lipid synthesis (↓ ceramides), weakening the barrier; ↑ IL-31 drives pruritus and the itch–scratch cycle. Dysbiosis (e.g., *S. aureus*) amplifies inflammation via superantigen/TLR pathways, further elevating cytokine release and sustaining chronic inflammation. Figure created by the authors.

AD is a disease of epidermal barrier dysfunction that is largely driven by structural and functional abnormalities in the stratum corneum (SC), the outermost layer of the epidermis. The SC functions as a physical and chemical barrier composed of a lipid matrix and anucleated corneocytes, which are terminally differentiated keratinocytes [12]. The stratum granulosum (SG), a layer found beneath the SC, functions as a secondary barrier by housing keratinocytes that are sealed together with tight junctions. The brick-and-mortar structure of the epidermis prevents transepidermal water loss (TEWL), regulates skin pH, and restricts the penetration of microbes, promoting microbial integrity of the skin. In AD, barrier integrity is compromised by reduced ceramide content, altered lipid organization, and defective cornification [13]. Therefore, skin barrier impairments result in TEWL, which explains the xerosis seen in patients with AD, as well as greater penetration of microbes and allergens, resulting in exacerbation of symptoms. Central to this dysfunction is the deficiency in a structural protein, filaggrin (FLG), encoded by the FLG gene located within the epidermal differentiation complex (EDC) on chromosome 1q21 [12]. FLG is produced through the proteolytic cleavage of profilaggrin and is critical for SC integrity. It aggregates with keratin filaments within corneocytes to provide a framework for the assembly of other structural proteins and is eventually broken down into natural moisturizing factors (NMFs) like pyrrolidone carboxylic acid (PCA) and urocanic acid (UCA), which help maintain hydration and acidify the skin surface [14]. Loss-of-function mutations in FLG are a major genetic risk factor for the development of AD, causing abnormal corneocyte morphology and reduced NMFs. Resultantly, weakened corneocyte adhesion, excessive water loss, and elevated skin pH levels impair barrier function and protease regulation and enhance susceptibility to *Staphylococcus aureus* colonization. FLG-related proteins like hornerin and filaggrin-2 are sources of small antimicrobial peptides that inhibit bacterial growth, especially in acidic conditions. In AD, higher skin pH limits the activity of these antimicrobial peptides, contributing to skin dysbiosis and sustained inflammation [14]. The pathophysiology of AD suggests a feed-forward loop where barrier disruption promotes inflammation via stimulation of pro-inflammatory cytokines and causes further structural breakdown. However, barrier dysfunction also occurs in the absence of FLG mutations due to cytokine-driven down-regulation of FLG and other EDC genes. Cytokines such as IL-4, IL-5, IL-9, and IL-13 can inhibit FLG, suggesting that inflammatory pathways can mimic genetic mutations [15]. Additionally, AD exhibits alterations in tight junction proteins (TJPs) such as claudin-1, which further weaken barrier function. TJPs reside below the SC and regulate paracellular water and solute flux; their dysfunction amplifies TEWL [13]. The defective epidermal barrier, a hallmark of AD, sets the stage for microbial invasion and immune activation, but it also poses a significant hurdle to topical drug absorption (Figure 1, Table 1).

**Table 1:** Summary of skin barrier defects in atopic dermatitis This table outlines the major structural and functional defects of the epidermal barrier found in atopic dermatitis (AD). Key features include reduced ceramide content, filaggrin (FLG) deficiency, altered lipid organization, tight junction protein abnormalities (e.g., claudin-1), increased transepidermal water loss (TEWL), elevated skin pH, microbial dysbiosis, and enhanced protease activity. These abnormalities contribute to impaired hydration, increased allergen penetration, and heightened inflammation. AD: atopic dermatitis; FLG: filaggrin; TEWL: transepidermal water loss; pH: potential of hydrogen (acidity/alkalinity measure)

Defect	Description	Pathophysiologic Impact
Reduced Ceramide Content	Lower levels of ceramides, key lipids in the stratum corneum	Weakens lipid barrier, increases transepidermal water loss (TEWL), impairs hydration, allows allergen penetration
Filaggrin Deficiency	Loss-of-function mutations or cytokine-induced downregulation of filaggrin (FLG)	Disrupts corneocyte structure, reduces natural moisturizing factors, and increases pH and dryness
Altered Lipid Organization	Disrupted arrangement of cholesterol, fatty acids, and ceramides in the stratum corneum	Impairs barrier integrity and drug penetration, heightens sensitivity
Tight Junction Protein Defects	Decreased expression/function of claudin-1 and other tight junction proteins	Weakens the secondary barrier, amplifies water loss, and allows allergen ingress
Increased TEWL	Excessive loss of water through the epidermis	Leads to xerosis, barrier breakdown, itch-scratch cycle
Elevated Skin pH	Loss of acid mantle due to reduced natural moisturizing factors and altered microbiome	Promotes pathogen colonization (e.g., *S. aureus*), impairs enzyme regulation, and increases inflammation
Microbiome Dysbiosis	Reduction in beneficial skin flora and overgrowth of pathogenic bacteria	Triggers inflammation, worsens barrier damage, and increases infection risk
Enhanced Protease Activity	Upregulation of proteases due to increased pH and inflammation	Degrades structural proteins, perpetuates barrier damage

Changes in lipid structure and irregular corneocyte layering lead to variable drug penetration and increased irritancy, ultimately affecting the efficacy of topical therapies [16]. Most topical drugs permeate along lipid regions, making the lipid organization of the skin barrier an especially essential component of its functionality [17]. The inflammatory nature of AD further complicates absorption by altering skin enzyme activity, which may chemically alter or degrade topical therapies, reducing their bioavailability. Elevated skin pH levels not only affect the passive diffusion of topical agents but also their stability. The composition of the topical agents applied plays a critical role. Hydrophobic formulas being delivered in traditional ointments may be poorly absorbed into the skin with AD due to the high variability in hydration. These challenges emphasize the need for drug delivery methods that are barrier-compatible and can optimize drug release while catering to the unique physiological environment of skin affected by AD.

Overview of transdermal delivery systems

The truly relentless pruritus, lesions, and flares associated with AD pose unique challenges. Improving transdermal drug delivery systems can allow for therapies to be safer and more targeted, and avoid adverse effects associated with traditional therapies such as corticosteroids [17]. While control of inflammatory mediators is the main goal, these topical delivery systems, especially in persons with compromised skin barriers, may not be able to absorb well or equally in the skin and create unintended effects like skin atrophy and striae [17,18]. Alternative delivery options like transdermal methods have the potential to be safer, more targeted, and bypass systemic side effects associated with certain steroid or immunosuppressive medications [19,20]. Natural diffusion of medications through the skin with lipophilic and hydrophilic properties is known as a passive strategy of drug delivery [21,22]. These drugs are largely very small, often being <500 Da, and delivery can be coupled with liposomes, hydrogels, or nanocarriers to facilitate uptake in the skin; however, the SC layer disallows passive delivery of larger molecules [20,22]. There has been significant improvement in drug delivery systems for conditions with greater variability in the quality of the skin barrier, such as AD. Use of active transdermal techniques, in contrast, uses external devices or energy to allow drugs to penetrate past the skin barrier [21]. Overcoming the skin barrier and employing techniques that aim for improved penetration of medications that may be limited by diffusion can avoid the limitations that skin barrier challenges pose. Iontophoresis utilizes currents to push charged drugs, sonophoresis employs the use of ultrasound wave technology, and the creation of micropores via high-voltage pulses through electroporation are all examples of active techniques that can improve drug penetration [21]. Transdermal patches have been explored as an alternative, utilizing electrospun nanofiber membranes to allow release of urea or pioglitazone [17]. These patches are theorized to allow for retention of delivered medication deep within the skin layers and allow a more controlled release of medication once applied [17,18]. Additionally, microneedle patches can allow for deposition of medication into deeper layers to allow for direct absorption [19]. In a mouse model with AD, microneedles constructed from dissolvable polymers demonstrated rapid drug release and biocompatibility, allowing for facilitated transdermal delivery of corticosteroids, leading to reduced inflammation and immune cell infiltration [19]. Targeted delivery through transdermal systems can be applied to a variety of immune-mediated skin disorders, allowing for local efficacy.

Vehicle formulations used in atopic dermatitis

Nanoscience innovations coupled with dermatology can make way for increased avenues to treat AD with increased efficacy, longer release of medication, and decreased systemic toxicities.

Emollient and occlusive vehicles

Management of AD can be done through modern emollients and occlusive vehicles. In order to restore the epidermal barrier and retain proper moisture, ingredients like ceramides and colloidal oatmeal can aid the efficacy of transdermal drug delivery [23]. Drug penetration while maintaining the sensitive existing barrier is needed while prioritizing safe formulation and skin compatibility. Additionally, delivery must be consistent across a diversity of skin types and textures [23]. Emollients can reduce AD-associated irritation by improving skin hydration and flexibility and filling the gaps between dehydrated corneocytes [24]. Lipid-rich occlusives, including lanolin, petrolatum, paraffin, and mineral oil, can form hydrophobic interactions over the skin surface. Creation of this barrier can allow for the prevention of TEWL [24]. One study synthesized evidence that colloidal oats act as an effective occlusive agent, forming a physical barrier preventing TEWL [25]. Furthermore, oats can promote humectant production, maintain an optimal skin pH, and have bioactive components like avenanthramides that can mitigate inflammation associated with barrier disruption and allergic sensitization [25]. Challenges lie in the ability to deliver therapeutic agents without exacerbation of existing barrier dysfunction. For example, while olive oil, a natural emollient, is useful for its anti-inflammatory nature, the oleic acid component can disrupt the organization of lipids in the SC layer, creating difficulty for barrier integrity [26]. Effective barrier maintenance through effective drug delivery methods can help increase SC hydration and allow for AD to be managed by capturing moisture.

Liposomal and nanocarrier systems

The usage of specifically targeted therapies to reach inflamed areas with precision led to a need for liposomal and nanocarrier systems. Active drug molecules can be distributed through small delivery vehicles such as liposomes, ethosomes, transfersomes, solid lipid nanoparticles (SLNs), and nanostructured lipid carriers (NLCs), as well as polymeric nanoparticles [27]. Spherical liposomes are able to transport both hydrophilic and lipophilic drugs within their membranes and seamlessly fuse into the SC [27]. Interactions between liposomes and the skin barrier are due to biomimicry, where the lipid composition of liposomes mimics epidermal ceramide, cholesterol, and fatty acid compositions as medications are delivered through the skin barrier. Use of liposomal formulations allows for enhanced drug deposition in the epidermal and dermal layers, leading to better absorption and retention [28]. These delivery systems can lower systemic absorption so that drug delivery can be specific to one location and off-target effects are reduced [17,28]. In AD, a number of investigational treatments utilizing liposomal delivery have primarily led to evidence showing encouraging outcomes. One prominent study involved the use of Janus kinase (JAK) inhibitors, which are typically administered orally or by injection. These can be highly effective in modifying immunological therapies in AD [17]. Researchers created a topical nanoliposomal emulsion containing the JAK1/2 inhibitor ruxolitinib and tested it on individuals with moderate AD. The nanoliposomal ruxolitinib gel dramatically reduced itching and eczema severity scores over the course of the four-week treatment [17]. Erythema was reduced at sites, and moisture was retained better where applied [17]. Evaluation of ceramide-enriched liposomes made of phosphatidylcholine, cholesterol, and ceramide allows for high skin permeation and strong drug retention [27]. These findings support that a stronger formulation and optimized delivery to skin with AD may occur when liposomes are composed with a higher concentration of skin lipids. Furthermore, increased malleability of liposomes and ethosomes can allow for increased penetration, while the use of solidifying lipid nanoparticles that become a solid state at room temperature can allow for extended release of medication over time [27].

Ethosomes are soft vesicular carriers containing high concentrations of ethanol (20-45%) that enhance deformability and skin penetration. Their ethanol content loosens intercellular lipid packing in the SC, improving drug flux deeper into the skin layers. Almuqbil et al. (2025) further discuss structural design and stability, emphasizing that ethosomes can adapt to different drug types (hydrophilic/lipophilic) with relative biocompatibility, increasing skin deposition while reducing systemic leakage [29]. In a BALB/c AD mouse model, a 0.1% piperine ethosomal cream reduced ear/skin thickness, clinical severity scores, leukocytes, granulocytes, and serum IgE significantly vs. vehicle and vs. a matched conventional piperine cream (0.125%) and showed greater overall efficacy than 0.1% tacrolimus ointment (head-to-head in the same model) [30].

Transfersomes are ultra-deformable vesicles (elastic liposomes) that incorporate edge activators, surfactants that increase membrane flexibility, allowing them to squeeze through narrow intercellular spaces in skin. This flexibility helps them traverse compromised or irregular skin barriers more effectively than conventional liposomes. While fewer studies address AD specifically, transfersomes have been used for enhanced transdermal delivery of drugs in inflammatory or barrier-impaired skin models, and their low irritation profile is often cited as an advantage. Notably, tacrolimus-loaded transfersomes showed improved dermal penetration and stronger therapeutic effects in AD models compared with standard ointments, and nano-transfersomal chitosan gels have since been developed to further improve stability and penetration [31,32]. In an AD mouse model, tacrolimus nano-transfersomes in chitosan gel (TRL-NTsG) formed uniform vesicles (~163 nm, ζ −27 mV) and delivered ~8-fold higher in-vitro release and ~6-fold higher ex-vivo permeation versus conventional tacrolimus gel; ear thickness fell to 0.6 mm (vs 1.7 mm untreated and 1.3 mm conventional gel), IgE decreased to 120 ng/mL, H&E showed no toxicity, and the formulation was stable for ≥6 months [33].

SLNs are lipid matrix nanoparticles that are solid at both room and body temperature, stabilized by surfactants, that mimic cutaneous lipids. Their occlusive properties allow moisture retention and enhanced epidermal drug partitioning. Several preclinical studies have explored SLNs as carriers for calcineurin inhibitors in AD. For example, tacrolimus-loaded SLN gels demonstrated particle sizes of 439-669 nm, polydispersity indices (PDI) ≤ 0.4, and entrapment efficiencies of 68-84%, with a pH of 5.45-5.53 suitable for skin application; incorporation into gel retarded drug release but significantly improved skin retention compared with SLNs alone [34]. Thermosensitive tacrolimus SLNs exhibited an amorphous drug state with high loading efficiency, and in ex vivo rat skin models, they achieved deeper dermal penetration than 0.1% Protopic® ointment [35]. In vivo, cyclosporine A (CsA)-loaded SLNs alleviated AD-like symptoms in murine models, reducing Th2 cytokines (IL-4, IL-5) and demonstrating ~2-fold higher skin permeation than CsA in oil mixtures [36]. Collectively, these results indicate that SLNs can enhance dermal delivery and epidermal retention of lipophilic immunomodulators while reducing systemic exposure. However, full AD lesion efficacy readouts (ear thickness, IgE, histology) for tacrolimus-SLN systems remain limited, highlighting the need for further translational and long-term safety studies.

NLCs build on SLNs by combining solid and liquid lipids, creating a less-ordered matrix that accommodates higher drug loading and reduces drug expulsion. This internal “imperfection” allows more stable entrapment and sustained release. Their use in inflammatory skin disease models has shown improved drug retention in target layers and lower systemic leakage. For example, in a CsA NLC dermal gel optimized for AD, the optimized batch reported a particle size of ≈ 266 nm and ζ ≈ −30 mV, a gel pH of 5.2-5.5, and complete in vitro drug release by six h. In AD-induced rat skin, ear-flap thickness decreased by ~2× vs. disease control, and no irritation was observed. Such systems may offer a balance between enhanced penetration and controlled retention, reducing systemic toxicity [35].

Polymeric nanoparticles are fabricated from biocompatible polymers (e.g., PLGA, chitosan), enabling controlled release and surface functionalization (targeting, charge tuning). Their rigid matrices allow more sustained delivery, but their penetration depth is limited without assistance (e.g., penetration enhancers). Recent reviews emphasize polymeric systems as promising for the delivery of macromolecules and peptides when combined with penetration strategies (e.g., surface charge, small size). In AD murine models, PLGA nanoparticles accumulated preferentially in inflamed skin, with ~15-fold higher deposition for 70 nm versus 300 nm particles, suggesting lesion-selective uptake [37,38]. Tacrolimus-loaded chitosan nanoparticles have also reduced inflammation and IgE levels, highlighting their potential to lower systemic dosing needs [39]. In AD, polymeric nanoparticles could thus localize biologics or small-molecule immunomodulators with minimized systemic exposure.

Hydrogel and microemulsion-based systems

Hydrogel- and microemulsion-based systems provide an attractive option for the transdermal delivery of therapeutics in AD, simultaneously addressing inflammation, hydration, and patient comfort [40]. Hydrogels are formed by three-dimensional networks of hydrophilic polymers, with the ability to accommodate significant amounts of water, at least 10% of the total material weight [41]. This water-rich material possesses a high degree of flexibility, mimicking the biomechanical properties of skin tissue closely. This same feature produces a cooling effect upon application, calming down pruritic and inflamed lesions, hallmark features of AD [42]. Furthermore, hydrogels act as an effective platform for uniform drug distribution along with controlled release, which is especially useful in pharmaceuticals such as tacrolimus or crisaborole, which require a consistent delivery onto the defective SC [43]. By contrast, microemulsions are thermodynamically stable colloidal systems composed of oil, water, surfactants, and cosurfactants. Their nanometer-sized dimensions enhance drug solubilization and cutaneous penetration, allowing easier passage through the disturbed lipid matrix of AD-affected skin [44]. According to a study by Shetty and Sherje, microemulsions can improve the dermal delivery of corticosteroids like betamethasone dipropionate, possibly allowing the reduced application of steroids in terms of frequency and quantity while still maintaining their therapeutic effect [45]. Together, hydrogels and microemulsions represent versatile, patient-centered delivery systems that hold significant promise in optimizing transdermal therapy for AD, particularly by enhancing efficacy while improving tolerability and adherence. Due to their characteristics of spreadability, non-greasy texture, and minimal staining residue, these microemulsion-based systems are better accepted by patients compared to traditional ointments. This is of utmost importance in the pediatric and adolescent population, where treatment adherence is most likely influenced by sensory appeal, such as texture and ease of application [46]. However, limitations persist. Certain microemulsions may contain surfactants that have the potential to cause irritation on already compromised and inflamed skin. Another consideration is formulation stability, which can be hard to maintain over prolonged periods of time [47]. Hydrogels, on the other hand, are better tolerated but may offer limited penetration in thickened lesions or severely lichenified areas, reducing their effectiveness in more advanced cases of AD. In addition to some anti-inflammatory agents, these systems are used in clinical practice as vehicles for antimicrobial agents (e.g., a hydrogel of fusidic acid). Thus, these agents would act dually in superinfected eczema [48]. Large trials are limited; however, phase 1 and 2 studies suggest that they are safe and effective. Future modifications may consider pH-responsive hydrogels or thermosensitive systems operating with heat from the skin or inflammation markers as triggers for drug release [48,49]. In general, hydrogel- and microemulsion-based systems provide for a patient-friendly, inflammation-targeted approach well aligned with the complex demands of transdermal therapy in AD.

Polymeric and film-forming systems

In addition to hydrogel- and microemulsion-based systems, polymeric and film-forming systems also offer a novel frontier in the treatment of AD, with a particular characteristic in accomplishing sustained drug delivery and improving patient adherence. These systems utilize polymers, either natural and/or synthetic, to form semi-occlusive films or adhesive patches with the ability to directly attach to the skin surface [50]. Once applied, these systems form a thin, protective film capable of producing a prolonged drug release while reducing contamination. This sustained release mechanism is especially useful in medications such as calcineurin inhibitors or low-potency corticosteroids, as it reduces the need for frequent reapplication [51]. This will result in greater patient adherence, especially in chronic conditions like AD. Film-forming systems have been shown to have better skin retention results and lengthened therapeutic effects compared to traditional topical creams and ointments. For example, studies of dermatological drug patches containing either mometasone furoate or hydrocortisone acetate prove the steady delivery of corticosteroids for 24 hours, which thereafter precludes the fluctuations in therapeutic levels [52]. Being occlusive in nature, the polymeric film also helps form the skin barrier against TEWL, which is considered the primary issue in AD pathology. From a pediatric standpoint, the patch systems constitute an advantage, as they require fewer doses and allow for clean, nongreasy applications that do not interfere with daily activities. For caregivers, the ability to apply to the child a patch or a film once per day or even less frequently will minimize treatment burden, an important consideration with childhood AD management [46]. Yet, no system is perfect, and some formulations may also cause mild irritation or discomfort upon removal; strong adhesion under active scratching, meanwhile, can be challenging, along with adhesive adherence on exudative lesions. Besides that, kids with sensitive skin can develop contact reactions to an adhesive ingredient, which may call for either patch testing or a reformulation [53]. The other developments being researched include biodegradable polymers, smart films responding to skin temperature or pH, and multilayers for combination drug delivery [54]. On the whole, the polymeric and film-forming systems are finally emerging as a feasible, patient-oriented solution aimed at simplifying therapy and enhancing the outcome of AD, particularly for populations struggling with adherence. Key characteristics and clinical considerations for vehicle types used in AD therapy are summarized in Table 2.

**Table 2:** Comparison of vehicle types used in atopic dermatitis therapy This table compares commonly used topical vehicle types for drug delivery in atopic dermatitis, highlighting their mechanisms, advantages, disadvantages, and clinical applications. Vehicle systems reviewed include emollients and occlusives, liposomal systems, nanoemulsions, hydrogels, polymeric/film-forming systems, solid lipid nanoparticles (SLNs), nanostructured lipid carriers (NLCs), and microemulsions. JAK: Janus kinase; NLCs: nanostructured lipid carriers; SLNs: solid lipid nanoparticles; AD: atopic dermatitis; pH: potential of hydrogen

Vehicle Type	Mechanism	Advantages	Disadvantages	Clinical Use/Notes
Emollients/Occlusives	Restore hydration, form a protective barrier	Improve moisture retention, affordable, and low irritancy	Greasiness, potential for contact allergy	Foundation of AD care; e.g., petrolatum, ceramide creams
Liposomal Systems	Encapsulate drugs in lipid vesicles	Enhance penetration, target delivery, mimic skin lipids	Stability issues, cost, and limited pediatric data	Nanoliposomal corticosteroids, JAK inhibitors
Nanoemulsions	Disperse the drug in nanoscale oil/water droplets	Improve solubility, uniform drug distribution	Surfactant irritation, stability concerns	Delivery of corticosteroids, antimicrobials
Hydrogels	Water-rich, polymeric networks	Cooling, soothing, non-greasy, uniform release	Limited penetration in thickened skin	Tacrolimus hydrogel, antimicrobial hydrogels
Polymeric/Film-Forming Systems	Form adhesive films/patches	Sustained release, improved adherence	Removal discomfort, adhesive reactions	Corticosteroid patches, calcineurin inhibitor films
Solid Lipid Nanoparticles/NLCs	Solid lipid matrix carriers	Prolonged release, enhanced stability	Manufacturing complexity, limited data	Delivery of anti-inflammatory drugs, ongoing trials
Microemulsions	Oil-water-surfactant-cosurfactant colloids	Enhanced penetration, rapid absorption	Surfactant irritation, short shelf-life	Betamethasone, anti-inflammatories

Clinical evidence and current applications

A growing body of literature supports the efficacy and tolerability of modern transdermal systems in the management of AD. Among the most established are emollient and occlusive vehicles such as ceramide-based creams, petrolatum, and colloidal oatmeal formulations. In a systematic review conducted by Hebert et al., it was found that nonprescription moisturizers with barrier-enhancing constituents significantly reduced SCORAD and itch severity in both pediatric and adult populations, without major adverse effects [23]. This suggests their continued role as foundational therapies for improving hydration and epidermal repair. Fowler et al. expanded on this by evaluating colloidal oats and confirmed their dual role as both humectants and anti-inflammatory agents, largely due to avenanthramides [25]. Furthermore, Kurebayashi et al. stressed that these vehicles aid drug penetration while minimizing irritation, providing even greater therapeutic value in children where skin integrity is fragile and sensitivity is high [24]. These findings emphasize hydration as a safe and consistent form of adjunct to any transdermal application strategy. More advanced approaches, such as liposomal and nanocarrier delivery systems, have demonstrated marked improvement in targeted drug delivery directly to inflamed tissue. Garrós et al. reported that baricitinib-loaded lipid nanosystems had greater epidermal retention and anti-inflammatory efficacy compared to conventional topical agents, both in in vitro and in vivo models [27]. This absorption pattern may be attributed to lipid mimicry between the carrier and SC, enabling deeper permeation in active lesions. Since pediatric trials remain sparse, early studies in adult AD patients suggest that nanocarriers can reduce the need for higher steroid doses by localizing drug action more effectively. Nevertheless, issues remain to be addressed in terms of formulation stability, carrier cytotoxicity, and scalability in production. Despite its chronic nature and pattern of localized flare-ups, adequate concentrations of therapy at the sites could reduce systemic exposure and minimize long-term risks. Hydrogel and microemulsion-based systems have shown promise in enhancing both comfort and adherence, particularly in pediatric and adolescent populations. A randomized controlled study involving 100 pediatric patients found that tacrolimus hydrogel significantly reduced inflammatory biomarkers and improved modified Eczema Area and Severity Index (EASI) scores more effectively than 1% hydrocortisone cream [23]. The hydrating and cooling nature of hydrogels appears to make them especially soothing for itchy, pruritic skin, a hallmark of AD. Likewise, microemulsions have shown enhanced corticosteroid delivery with fewer applications. Shetty and Sherje’s study found that betamethasone dipropionate in a microemulsion base achieved similar clinical clearance to standard formulations but with a 40% reduction in frequency of application and greater patient satisfaction [45]. These advantages are particularly critical in children, where non-greasy, fast-absorbing textures directly influence adherence. Still, surfactants in some microemulsions can irritate compromised skin, limiting their use in severe flares unless formulations are optimized for sensitivity. Finally, polymeric and film-forming systems have emerged as novel vehicles offering sustained drug release and better therapeutic consistency. Recent investigations into corticosteroid patches, such as mometasone furoate and hydrocortisone acetate, have shown these systems can maintain effective drug levels over 24 to 48 hours, decreasing the burden of frequent reapplication [24,27]. A 2024 study found that children using film-forming patches had improved symptom control and better caregiver-reported adherence compared to cream-based regimens [25]. The ability to "set and forget" a treatment patch can drastically improve the quality of life for both pediatric patients and their families. However, mild adhesive-related irritation was noted in about 10% of pediatric cases, underscoring the need for hypoallergenic formulations. Smart polymers responsive to pH or temperature could personalize drug release even further, but such innovations remain in early development stages. Overall, these technologies bridge a critical gap in chronic care by aligning therapeutic efficacy with patient usability. Together, these studies reinforce that delivery systems matter just as much as the active agents themselves in treating AD (Table 3).

**Table 3:** Recent preclinical studies of transdermal vehicles in atopic dermatitis (2020–2025) This table summarizes key preclinical studies published between 2020 and 2025 investigating advanced transdermal drug delivery vehicles for atopic dermatitis (AD). Included are study designs, vehicle types, experimental models (in vitro, ex vivo, in vivo), and principal findings on efficacy, skin penetration, and safety. Studies involve both animal and human cell models as indicated. AD: atopic dermatitis; CNPs: chitosan-based nanoparticles; DNFB: 2,4-dinitrofluorobenzene (AD-inducing agent); DNCB: 1-chloro-2,4-dinitrobenzene (AD-inducing agent); ex vivo: outside the living organism but in an isolated tissue (e.g., rat/porcine/human skin in diffusion cells); HaCaT: human keratinocyte cell line; IFN-γ: interferon gamma; IL: interleukin; LCNPs: lyotropic liquid crystalline nanoparticles; NEG: nanoemulsion gel; NLCs: nanostructured lipid carriers; NPs: nanoparticles; OVA: ovalbumin (used for inducing AD in animal models); SLNs: solid lipid nanoparticles; TAC: tacrolimus; TNF-α: tumor necrosis factor alpha; in vivo: within the living organism (e.g., animal models); in vitro: in cultured cells or controlled laboratory environment

Study (Year)	Vehicle Type	Model/Subjects	Key Findings/Outcomes
Lin et al., 2023 [55]	Niosomes	Ex vivo: Rat abdominal skin mounted on Franz diffusion cells	Optimized niosomes enhanced cutaneous drug retention in epidermal and dermal layers. Sustained drug release and effective stratum corneum interaction facilitated enhanced local delivery.
Xia et al., 2024 [56]	Liposome-based hydrogel	In vitro: HaCaT (human keratinocyte) cells. In vivo: AD model in rats induced by 1-chloro-2,4-dinitrobenzene (DNCB)	Improved skin permeability and sustained drug release. Promoted wound healing and reduced skin lesions in the rat AD model.
Huang et al., 2025 [57]	Polysaccharide-based nanoparticles	In vitro: Human keratinocyte cells (HaCaT). Ex vivo: Male rat skin mounted on Franz diffusion cells. In vivo: Male rats induced with AD using 2,4-dinitrofluorobenzene (DNFB)	Enhanced cellular uptake and anti-inflammatory effects, significantly reducing IL-6, IL-8, TNF-α, and IFN-γ in HaCaT cells. In vivo, the nanoparticles significantly reduced skin lesion scores, skin thickness, IL-4 and IgE levels, and mast cell infiltration. Demonstrated superior cutaneous delivery and immunomodulatory effect.
Rapalli et al., 2023 [58]	Lyotropic liquid crystalline nanoparticles (LCNPs)	In vitro: Human keratinocyte cells (HaCaT). Ex vivo: Excised rat skin mounted on Franz diffusion cells	LCNP gel demonstrated prolonged drug release, enhanced skin permeation, and superior retention in the epidermis and dermis compared to plain gel. In vitro model showed improved therapeutic efficacy.
Javia et al., 2022 [59]	Liposomes incorporated into Carbopol 934P gel	In vivo: BALB/c atopic dermatitis mice models induced by ovalbumin (OVA). Ex vivo: Mouse skin mounted on Franz diffusion cells	Exhibited superior skin permeation and retention compared to Omiganan gel. Significantly reduced pro-inflammatory cytokines in AD models.
Ibrahim et al., 2024 [60]	Mirtazapine-loaded invasomal gel (inva-gel)	Ex vivo: Excised rabbit ear skin mounted on Franz diffusion cells. In vivo: Male rats induced with AD using 2,4-dinitrochlorobenzene (DNCB)	Ex vivo studies showed enhanced drug permeation from inva-gels vs. control gels. In vivo application on rats significantly reduced dermatitis severity scores, scratching behavior, and epidermal thickness. Decreased mast cell infiltration and downregulation of inflammatory mediators.
Zhu et al. (2022) [61]	Zein–silk sericin nanoparticles (NPs)	In vitro: TNF-α/IFN-γ-stimulated HaCaT keratinocytes. Ex vivo: Porcine skin Synthetic Strat-M membrane in Franz diffusion cells	Confocal microscopy confirmed deeper dermal penetration of NPs. In vitro, NPs significantly reduced inflammatory cytokines (e.g., IL-6, RANTES, TARC) compared to free curcumin.
Xu et al. (2024) [62]	Nanoemulsion gel (NEG)	In vivo: Male rats induced with AD using 2,4-dinitrofluorobenzene (DNFB)	NEG reduced epidermal thickness, mast cell infiltration, and histopathological skin damage. Treatment suppressed serum and skin levels of IgE, TNF-α, IL-4, and IFN-γ.
Atmakuri et al. (2023) [63]	Nanoemulsion gel (NEG)	In vivo: AD model in mice induced by 1-chloro-2,4-dinitrobenzene (DNCB)	NEG showed significant retention in the stratum corneum and reduced transdermal permeation compared to plain gel, suggesting effective localization. NEG led to a significant reduction in dermatitis severity scores and pro-inflammatory cytokines (IL-6 and TNF-α). Demonstrated reduced epidermal thickening, inflammatory infiltration, and promoted skin healing.
Lee et al. (2024) [64]	Chitosan-based nanoparticles (CNPs) loaded with tacrolimus (TAC)	In vitro: Human keratinocytes (HaCaT cells), Mouse fibroblast cells (NIH3T3), Full-thickness human cadaver skin. In vivo: Mouse model of atopic dermatitis (AD)	The formulation showed sustained drug release and 4-week colloidal stability. CNPs at 1/10th the dose of commercial Protopic ointment produced similar anti-inflammatory effects, including reduced ear thickness and cytokine levels.

Across the spectrum, from emollients to nanocarriers, individualized treatment matching patient age, disease severity, and lifestyle may enhance long-term disease control. While pediatric safety data are strongest for traditional vehicles and hydrogels, early evidence supports cautious optimism for broader implementation of advanced systems. Further large-scale, longitudinal trials are needed to confirm safety and comparative effectiveness, but current findings suggest that these innovations hold promise for addressing the complex needs of AD management across the lifespan.

Challenges and limitations

To optimize formulations of occlusive vehicles, it is necessary to understand the balance between occlusiveness, patient compliance, and interactions with pharmaceutical ingredients. Current studies suggest that the variability in transdermal drug penetration can depend on the severity of AD. As AD progresses, the skin barrier becomes more impaired while TEWL becomes elevated, altering drug absorption patterns [65-67]. A 2016 animal model study found a 2.7-fold increase in the cutaneous deposition of lipophilic tacrolimus in AD-like skin when compared to more intact skin. It was also reported that hydrophilic drugs, such as methotrexate and dextran, demonstrated an approximately 18-fold higher transdermal flux in AD-like skin [68]. Other studies have demonstrated that the skin of patients with AD exhibits higher permeability to transdermal drugs in comparison to healthy skin. For example, in a 2017 systematic review, it was reported that patients with AD experience approximately double the rate of skin absorption of penetrants, such as medications [66]. These findings imply that the degree of disease severity, which is characterized by barrier disruption and inflammation, may influence the penetration of transdermal drugs. As this relationship is nuanced, further studies are needed to examine how specific skin structures and drug properties play a role in drug penetration.

Beyond efficacy, the long-term safety of novel vehicles on eczematous skin warrants explicit attention. Diseased epidermis demonstrates materially higher permeability than healthy skin (often on the order of ~2×), raising the possibility of greater systemic exposure during chronic use. In ex vivo human tissue, Bozorg et al. detected hydrocortisone in the receptor chamber after passive application to eczematous/psoriatic skin, consistent with enhanced permeation across a compromised barrier with lower electrical resistance, alongside disease-related changes in TEWL and surface pH that track with increased penetration [67]. These considerations extend to penetration enhancers and innovative carriers: while they improve delivery, repeated use on inflamed or eroded sites may increase irritation and cumulative systemic exposure. Accordingly, future AD trials of transdermal systems should prespecify pharmacokinetic monitoring (e.g., trough concentrations for chronically used actives), report exposure-over-time data, include pediatric cohorts, and evaluate chronic-use tolerability on lesional vs. non-lesional skin. These data are prerequisites for regulatory assessment of benefit-risk in long-duration, real-world therapy.

Furthermore, evidence on novel drug delivery vehicles and their long-term safety and exposure remains limited. This gap demonstrates concerns regarding the long-term treatment of AD with these novel vehicles and the potential adverse effects, limiting clinical translation. While these transdermal vehicle innovations improve drug solubility and enhance skin penetration, their introduction into clinical practice is hindered by multifaceted barriers. For instance, novel vehicles may still be subjected to extensive evaluation for approval, despite the active pharmaceutical ingredient being approved. With the lack of comprehensive, long-term safety data and regulatory guidelines for these vehicles, the clinical translation and approval process are further complicated [69]. Similarly, cost remains another barrier. The manufacturing processes and technological development associated with these novel vehicles significantly increase the costs of production when compared to conventional formulations, and consequently, the economic burden may be placed on patients as well [70]. As a result, the clinical utility of these transdermal vehicle innovations remains underused, despite their potential to improve treatment outcomes.

Future directions

The continued advancement of transdermal drug delivery systems (TDDS) in AD offers a promising path toward more effective and patient-centered care. One key future direction is the personalization of TDDS based on individual skin barrier characteristics. As barrier integrity in AD varies due to factors such as filaggrin mutations, inflammation, and regional skin differences, tailoring drug vehicles using non-invasive measures like TEWL or lipid profiling may enhance therapeutic precision and reduce irritation [14]. Smart, stimuli-responsive delivery systems also represent an exciting area of innovation. Vehicles that release drugs in response to changes in pH, temperature, or inflammation could enable more targeted treatment during disease flares, minimizing unnecessary drug exposure and improving disease control [71-73]. These technologies are particularly promising for pediatric patients or those with fluctuating symptoms, where adherence and tolerability are key concerns. Artificial intelligence (AI) is also emerging as a powerful tool to complement these innovations. Machine learning models are increasingly capable of integrating clinical records, genetic data, microbiome composition, and skin imaging to stratify patients, predict treatment responses, and inform personalized therapy [20]. AI-based diagnostic tools have demonstrated high accuracy in lesion classification and severity scoring, offering more objective and reproducible assessments than traditional clinical grading [20]. In parallel, AI is accelerating drug discovery by analyzing gene expression signatures to identify therapeutic targets and generate novel compounds structurally similar to approved AD treatments [20,74]. Additionally, real-time monitoring tools, such as wearable sensors that track pruritus and sleep, can be linked to AI algorithms to dynamically assess treatment efficacy and support timely therapeutic adjustments [75]. These technologies hold significant potential to enhance both the development and delivery of individualized transdermal therapies. To realize the full clinical potential of these emerging systems, robust clinical trials will be essential. Most current studies are limited by short durations, small sample sizes, or a lack of pediatric data. Future research should prioritize long-term safety, standardized outcome measures, and diverse populations to better inform real-world applications. Widespread adoption will also depend on addressing regulatory, manufacturing, and economic challenges [69,70,72]. Although many innovations build upon existing pharmacologic agents, novel delivery vehicles often face separate approval hurdles. Scalable production methods, clear regulatory guidance, and health economic evaluations will be crucial for ensuring accessibility. In defining the future niche for TDDS, it is important to contextualize them relative to systemic biologics, which currently represent the most effective long-term therapy for moderate-to-severe AD. Biologics such as dupilumab have demonstrated superior efficacy and favorable risk-benefit profiles compared with traditional immunosuppressants; however, phase III trials show that only about one-third of patients achieve IGA 0/1, and many fail to reach complete clearance [76,77]. This highlights the need for additional innovative therapies. Seen in this way, TDDS may serve as complementary or alternative strategies by delivering drugs locally within the skin, thereby limiting systemic exposure while addressing key drawbacks of biologics, including cost, injection burden, and potential systemic adverse effects.

## Conclusions

Innovations in TDDS are redefining the therapeutic landscape for AD, a condition driven by chronic inflammation and disrupted skin barrier function. As reviewed in this manuscript, advanced vehicles, such as liposomes, nanoemulsions, hydrogels, and polymeric films, demonstrate significant potential to improve drug absorption, reduce irritation, and support long-term disease control. These technologies are specifically engineered to overcome the unique permeability challenges of AD skin while prioritizing patient-centered outcomes such as comfort, adherence, and safety. The emergence of stimuli-responsive carriers and the integration of artificial intelligence into diagnostic, monitoring, and drug development tools further expand the promise of individualized care. These technologies enable more precise, adaptive treatment strategies that align with the clinical and biological variability observed in AD. Despite this progress, key challenges remain. There is a critical need for standardized, longitudinal clinical trials to evaluate the long-term safety, efficacy, and real-world impact of novel delivery systems. In parallel, regulatory clarity, scalable manufacturing, and integration into clinical workflows will be essential for translating innovation into accessible care.
